# Intensive care unit acquired weakness: A protocol for an overview of systematic reviews and meta-analysis

**DOI:** 10.1097/MD.0000000000021926

**Published:** 2020-08-21

**Authors:** Zheng Li, Yitong Cai, Qian Zhang, Peng Zhang, Ruixiang Sun, Haijiao Jiang, JingJing Wan, Fang Wu, Xiaoye Wang, Xiubin Tao

**Affiliations:** a Graduate College, Wannan Medical College, Wuhu; b School of Nursing, Lanzhou University, Lanzhou; c The First Affiliated Hospital of Wannan Medical College, Wuhu, China.

**Keywords:** assessment of multiple systematic reviews-2, grading of recommendations assessment, development, and evaluation, intensive care unit-acquired weakness, overview, preferred reporting items for systematic reviews and meta-analyses statement

## Abstract

**Background:**

Intensive care unit-acquired weakness (ICU-AW) is an acquired neuromuscular lesion and a common occurrence in patients who are critically ill. There are already systematic reviews on ICU-AW. Therefore, we provide a protocol for an overview of systematic reviews to improve the effectiveness of the construction of an evidence-based practice for prevention of ICU-AW.

**Methods:**

We will search the PubMed, CINAHL, EMBASE, and the Cochrane Library for the relevant systematic review or meta-analyses about ICU-AW. Study selection, data extraction, and the quality assessment of the included studies will be performed independently by 2 reviewers. And the methodological quality, report quality and evidence quality will be evaluated by Assessment of Multiple Systematic Reviews-2 tool, Preferred Reporting Items for Systematic Reviews and Meta Analyses Statement checklist and Grading of Recommendations Assessment, Development and Evaluation system, respectively.

**Results:**

This overview of systematic reviews and meta-analysis will collect the evidence published about the ICU-AW.

**Conclusion:**

We hope that our research will contribute to clinicians and public decision making about the ICU-AW.

**Registration number:**

INPLASY202070067

## 1 Introduction

Intensive care unit-acquired weakness (ICU-AW) is an acquired neuromuscular lesion and a common occurrence in critically ill patients.^[1]^ The incidence is 25% to 85%,^[2]^ and it is still as high as 36% after discharge.^[3]^ Neuromuscular weakness in the ICU is most often due to critical illness myopathy (CIM), critical illness polyneuropathy (CIP), or critical illness neuromyopathy (CINM) a combination of the two.^[4]^ ICU-AW can prolong the patient's mechanical ventilation time and ICU hospital stay, increase the mortality rate and affect the patient's survival rate and quality of life after discharge.^[5]^

The most common form of ICU-acquired myopathy is CIM,^[6]^ CIM often begins within several days of intensive care unit (ICU) admission.^[7]^ The most common clinical manifestations of CIM are flaccid quadriparesis that may affect proximal more than distal muscles and failure to wean from mechanical ventilation.^[8,9]^ CIP usually occurs in patients who are in the ICU for 1 or especially 2 weeks or more.^[10,11]^ Cardinal clinical manifestations include limb muscle weakness and atrophy, reduced or missing deep tendon reflexes and loss of peripheral sensation to light touch and pinprick.^[12]^ CIM is usually reversible over weeks to months, but leads to prolonged intensive care unit (ICU) stays and increased length of hospital stay overall.^[13,14]^ Patients with CIM tend to have better outcomes than those with CIP. In survivors of CIP with mild or moderate nerve injury, recovery of muscle strength generally occurs over weeks to months.^[15,16]^ CINM has clinical features that overlap the individual but closely corresponding features of CIM and CIP, with symmetric weakness of all 4 limbs, typically affecting proximal more than distal muscles; reduced or absent deep tendon reflexes; and peripheral sensory loss.^[17]^

Neuromuscular weakness due to critical illness myopathy (CIM) or critical illness polyneuropathy (CIP) is a common occurrence in patients who are critically ill, developing in ≥25 percent of patients who are mechanically ventilated in the intensive care unit (ICU) for at least 7 days.^[18,19]^ Risk factors include sepsis,^[20]^ multiorgan failure, paralytic agents and the systemic inflammatory response syndrome (SIRS).^[21,22]^

An overview of reviews is a comprehensive research method that comprehensively collects relevant systematic reviews of treatment, etiology, diagnosis and prognosis of the same disease or the same health problem.^[23]^ The systematic review integrates the results of multiple high-quality clinical studies, and its credibility is higher. Overviews conduct systematic review evidence from a higher level, the information contained is larger and more comprehensive, and the clinical practicality is stronger. It is particularly useful for informing health service policy and delivery.^[24,25]^

There are already systematic reviews on ICU-AW, including the risk factors and intervention methods of ICU-AW. When there are many relevant systematic reviews, the use of systematic review and re-evaluation to summarize evidence can improve the effectiveness of the construction of an evidence-based practice for prevention of ICU-AW. This study summarizes the systematic review/meta-analysis related to ICU-AW and conducts an overview of reviews.

## 2 Methods and analysis

### 2.1 Registration

This systematic review protocol followed the guidelines of Preferred Reporting Items for Systematic Reviews and Meta Analyses (PRISMA-P).^[26]^ This overview has been registered on the International Platform of Registered Systematic Review and Meta-analysis Protocols (INPLASY). The registration number is INPLASY202070067 and the DOI is 10.37766/inplasy2020.7.0067.

### 2.2 Eligibility criteria

We will include studies that met the following criteria: (1) published systematic review or meta-analysis; (2) ICU patients with no restriction on age and gender; (3) The intervention measures are early activity, physical rehabilitation, drug therapy, etc.; (4) ICU-AW related risk factors, assessment and diagnosis, interventions and prevention will be included. We did not limit the language or year of publication. We will exclude protocols, editorials, meeting abstracts, and other reviews.

### 2.3 Search methods for identifying the studies

#### 2.3.1 Electronic sources

PubMed, CINAHL, EMBASE, and the Cochrane Library will be searched from the inception to August 2020. The search strategies were developed by ZL and guided by XBT, who is an experienced evidence-based medicine researcher. The search terms were “intensive care unit – acquired weakness”, ““ICUAW”, ““ICU-AW”, “critical illness myopathy”, “critical illness polyneuropathy”, “critical illness neuromuscular abnormality” and “Meta-Analysis”, “systematic review”, “evidence synthesis”, “systematic literature review”. Relevant overview of systematic reviews and meta-analysis will also be searched to identify potential studies.

#### 2.3.2 Study records

EndNote X9 will be used to manage the initial search records. Two reviewers (ZL and QZ) will independently review the titles and abstracts based on the inclusion criteria. We will download the texts of the potential records to review them for inclusion further. Disagreements will be resolved by discussion or through consultation with a third reviewer (XBT). Study selection will be summarized in a Preferred Reporting Items for Systematic Reviews and Meta-Analyses (PRISMA) flow diagram.

#### 2.3.3 Data extraction and management

Two reviewers (ZL and YTC) will extract date independently by each reviewer using a standardized data collection form. We will collect the following date including: the name of the first author, publication year, study location, the main topic, type of included studies, number of included studies, number of participants, ICUAW incidence, quality assessment tools, the main outcomes and etc.

### 2.4 Quality evaluation

#### 2.4.1 Assessment of methodological quality of included reviews

Two reviewers (ZL and YTC) will independently assess the quality for each study by using the Assessment of Multiple Systematic Reviews-2 (AMSTAR-2) tool.^[27]^ The AMSTAR-2 tool is a modified version of AMSTAR that fits more closely the systematic reviews that include both RCTs and NRCTs. Two reviewers (LZ and PZ) will rate the quality of each meta-analysis as high, moderate, low and critically low based on the overall score of the AMSTAR-2. Conflicts between reviewers will be resolved through discussion and involving experts.

#### 2.4.2 Report quality of included reviews

We will also use PRISMA checklist^[28]^ to assess the report quality of the included reviews. The PRISMA statement for reporting quality consists of a 27-item checklist and a 4-phase flow diagram. When the literature score is 21 to 27, the report is considered relatively complete; when the score is 15 to 21, the report is considered to have some defects; when the score is less than 15, it is considered that there are relatively serious information defects.

#### 2.4.3 Quality of evidence of included reviews

We will rate the evidence as “high”, “moderate”, “low”, or “very low” in a conclusive table using the Grading of Recommendations Assessment, Development and Evaluation system.^[29]^

### 2.5 Statistical analysis

#### 2.5.1 Data synthesis

A descriptive synthesis of assessed systematic reviews is planned. The effect sizes from the meta-analyses will be presented as mean differences (WMD), standardized mean differences (SMD), odds ratios (OR), relative risks (RR) or risk differences (RD), depending on the data reported by the authors. In addition, whenever possible, the results will be reported with 95% confidence intervals (95% CI). Excluded papers will be listed, with reasons for exclusion stated.

#### 2.5.2 Assessment of heterogeneity

We can reflect the feasibility of meta-analysis by evaluating the heterogeneity of the included studies.^[30]^ According to the guideline of Cochrane Handbook, heterogeneity between RCTs can be quantified using I-square (*I*^2^) values, if *I*^2^ > is 50%, significant heterogeneity is considered, then a subgroup analysis is needed to determine the source of heterogeneity. If there is missing data in the included study, we will contact the author by email or phone to get the missing data. We will use Egger test to evaluate publication bias and small-study effect, and a *P* value < .1 in the test confirms the bias and small-study effect.^[31]^

## 3 Discussion

ICU-AW has high prevalence and always accompany with poor clinical outcomes. This overview of systematic reviews and meta-analysis will collect the evidence published about the ICU-AW. We hope that our research will contribute to clinicians and public decision making.

## Author contributions

XBT conceived the idea of research. ZL, YTC, and QZ developed the first draft of the manuscript. PZ, RXS, HJJ, JJW, FW, and XYW revised several versions of the manuscript.
